# The association between traffic safety health beliefs and traffic risk behaviors in secondary technical vocational school students in China

**Published:** 2019-08

**Authors:** Fenfen Li, Shumei Wang

**Affiliations:** ^*a*^Key Laboratory of Public Health Safety, Ministry of Education-Department of Children and Adolescent, Health, School of Public Health, Fudan University, Shanghai (200032), China.

## Abstract

**Background:**

Understand the traffic health beliefs and traffic risk behaviors among secondary technical vocational school students, explore the relationship between health beliefs and traffic risk behaviors, and to provide proof and direction for further traffic injury intervention.

**Methods:**

A cluster sampling method was used to conduct a questionnaire survey of all grade one and grade two students in a secondary technical vocational school in Jiangsu Province in April 2018, to investigate student’ traffic risk behaviors and traffic health beliefs. Behavior number was calculated according to the kinds of risk behaviors, and behavior score was calculated according to behavior frequency.

**Results:**

A total of 1079 students were investigated. Traffic risk behaviors were highly prevalent, including walking through a red light (24%), not using the crosswalk (22%), no using a helmet (69%), no using reflective equipment at night (78%). There were significant correlations among the traffic risk behaviors (P<0.001). Of the traffic risk behaviors in the past 30 days, 64% of the students had 3 or over, and 51.5% scored 6 or over. The total health belief score was 3.39±0.74, and for the 7 dimensions, “perceived susceptibility”, 3.68±1.00; “perceived severity”, 3.73±0.98; “perceived benefits”, 3.73±0.96; “perceived barriers”, 3.19±1.04; “cues to action”, 3.10±1.05; “self-motive”, 3.38±1.04; “self-efficacy”, 2.60±0.77. The results of GLM (generalized linear model) analysis showed that, “cues to action” and “self-efficacy” significantly affect both traffic risk behavior number and score, with OR of 1.60 (1.16-2.20) and 0.45 (0.33-0.61) for behavior number, and 1.65 (1.07-2.55) and 0.41 (0.27-0.62) for behavior score. “Cues to action” increase the incidence of traffic risk behaviors, which may reflect the fact that peer effect in vocational school students played significant role in traffic safety. “Self-efficacy” decrease traffic risk behaviors and other dimensions of health belief model had no significant effect, which Indicate further intervention direction for traffic safety education.

**Conclusions:**

Traffic risk behaviors in secondary technical vocational school students were highly prevalent and clustered. Peer effect in vocational school students should be taken into full consideration to improve traffic safety. Further intervention for traffic safety should increase health education to increase students’ health belief.

**Keywords:**

Secondary technical vocational school, Traffic injury, Risk behavior, Health belief

